# Real-world safety of catheter ablation for atrial fibrillation with contact force or cryoballoon ablation

**DOI:** 10.1007/s10840-020-00734-w

**Published:** 2020-05-11

**Authors:** Andrea Natale, Sanghamitra Mohanty, Laura Goldstein, Tara Gomez, Tina D. Hunter

**Affiliations:** 1 Texas Cardiac Arrhythmia Institute, St. David’s Medical Center, Austin, TX USA; 2 Scripps Clinic Division Interventional Electrophysiology Address, San Diego, CA USA; 3 Metro Health Medical Center, Case Western Reserve University School of Medicine, Cleveland, OH USA; 4 Department of Internal Medicine, Dell Medical School, Austin, TX USA; 5 Health Economics and Market Access, Biosense Webster, Inc., Irvine, CA USA; 6 CTI Clinical Trial and Consulting Services, 100 East RiverCenter Boulevard, Covington, KY 41011 USA

**Keywords:** Atrial fibrillation, Catheter ablation, Radiofrequency ablation, Contact force, Cryoablation, Safety

## Abstract

**Purpose:**

Real-world data can help medical administrators, physicians, and payers make evidence-based decisions regarding treatment choices. The objective of this study was to compare real-world safety outcomes with the latest catheter technologies used for the treatment of atrial fibrillation (AF).

**Methods:**

The Vizient Health Systems database, a large US hospital database, was used to compare acute complications in AF ablation with the contact force sensing THERMOCOOL SMARTTOUCH® Catheter or the THERMOCOOL SMARTTOUCH® SF Catheter (ST) versus the second-generation Arctic Front Advance™ Cryoablation Catheter (CB2) between September 2015 and June 2017. The primary outcome was a composite safety endpoint of acute ablation-related complications defined via ICD-10 diagnosis and procedure codes, including tamponade and other pericardial events, respiratory complications, stroke, cerebral or pre-cerebral occlusion/stenosis without infarction, vascular access complications, hemorrhage, phrenic nerve injury, myocardial infarction, and pulmonary embolism.

**Results:**

In total, 1473 ablations met all inclusion criteria (407 ST, 1066 CB2). Ablations for paroxysmal AF (PAF) had a lower complication rate than ablations for persistent AF (PsAF) (6.1% vs. 7.3%), as did ablations with ST compared with CB2 within each AF type (PAF 6.0% vs. 6.1%, PsAF 6.3% vs. 7.8%). Neither ablation catheter nor AF type was statistically significant after controlling for site volume, patient age, and comorbid conditions (ST vs. CB2: OR 0.86, *p* = 0.5544; PsAF vs. PAF: OR 1.08, *p* = 0.7376).

**Conclusion:**

Acute ablation-related complication rates were low and were not significantly associated with catheter technology. Increased risk of complication was attributable to low-volume sites and baseline patient characteristics.

## Introduction

The projected total cost of medical and indirect treatment for AF in 2019 is $35.7 billion according to a 2016 report commissioned by the American Heart Association [1]. Both monetary and societal cost associated with AF can be minimized by utilizing the safest and most effective treatments, but technological advances frequently outpace the evidence that is needed for informed decision-making.

Radiofrequency (RF) and cryoballoon (CB) ablations are well-established treatment modalities for AF ablation, and both are used frequently in paroxysmal (PAF) and persistent (PsAF) populations. Improvements in outcomes have been reported for both modalities with the introduction of the latest generations of each technology—contact force (CF) sensing RF ablation catheters and second-generation cryoballoon catheters [2–5]. Little published evidence exists currently that specifically compares the most recent products. Much of what does exist are single center studies that may not be representative and that lack sufficient power to detect potential differences in complication rates [6–10].

The objective of the current study was to compare acute procedure-related complications in a large population of real-world AF patients who underwent AF ablation with a contact force sensing THERMOCOOL SMARTTOUCH® Catheter or THERMOCOOL SMARTTOUCH® SF Catheter (ST; Biosense Webster, Irvine, CA) or a second-generation Arctic Front Advance™ Cryoablation Catheter (CB2; Medtronic, Minneapolis, MN), representing clinical practices across the USA. The results of this study are intended to provide real-world evidence to help medical administrators, physicians, and payers make informed decisions regarding the latest catheter technologies.

## Methods

### Study population

This retrospective population-based study used the de-identified Vizient Health Systems database of real-world inpatient and hospital-based outpatient billing records, which include records for insured and uninsured healthcare recipients from over 400 hospitals in 42 states. Both inpatient and hospital-based outpatient visit information was submitted by 98% of providers. This database includes patient demographics, hospital characteristics, complete diagnosis and procedure coding histories, and chargemaster billing records. All data used to perform this analysis were de-identified and accessed in compliance with the Health Insurance Portability and Accountability Act. As a retrospective analysis of a de-identified database, the research was exempt from IRB review under 45 CFR 46.101(b)(4).

Inclusion criteria for ablations comprising this study data were a procedure code for AF ablation coupled with a primary diagnosis of AF occurring between September 2015 and June 2017 in patients ≥ 18 years old. Ablation cases that included a concomitant atrioventricular node ablation, valve replacement or repair, cardiac implant, surgical cardiac ablation, or surgical left atrial exclusion procedure were excluded. If a patient had multiple AF ablations meeting all inclusion and exclusion criteria to this point, only the first ablation was included. From within the population meeting these criteria, cohorts were defined based on catheter descriptions in the chargemaster billing records. Thus, ablation procedures that did not use an ST or CB2 catheter, or that had insufficient catheter descriptions to determine the type of ablation catheter used, were excluded. Finally, the population was restricted to patients for whom AF type was recorded so that the cohorts could be further divided into PAF and PsAF subgroups. The latter restriction was an important component of the study design because AF type is believed to potentially be associated with both risk level and catheter choice.

### Patient characteristics

In addition to identifying the population with AF and the subgroups with PAF and PsAF, diagnosis codes were used to identify clinically important risk factors, including the presence of additional cardiac arrhythmias, risk factors comprising the CHA_2_DS_2_-VASc stroke risk score for AF patients, and available components of the HAS-BLED risk score for major bleeding. The CHA_2_DS_2_-VASc score was calculated by adding one point each for the presence of congestive heart failure (CHF), hypertension, age 65–74 years, diabetes mellitus, vascular disease, and female gender and two points each for history of stroke/transient ischemic attack (TIA) or age ≥ 75.

### Ablation procedures and complications

Ablation procedures using ST or CB2 catheters were identified via a text mining algorithm applied to the chargemaster descriptions and categories within the Vizient Health Systems real-world database. Ablations using both ST and CB2 catheters were assigned to the CB2 cohort in order to classify them based on the catheter most likely used for PV isolation.

The primary outcome was a composite endpoint of acute ablation-related complications recorded at any time during the ablation or prior to hospital discharge. These complications included tamponade and other pericardial events, respiratory complications, stroke, cerebral or pre-cerebral occlusion/stenosis without infarction, vascular access complications, hemorrhage and/or blood transfusion, phrenic nerve injury, myocardial infarction (MI), and pulmonary embolism (PE).

All diagnoses and procedures used to define the inclusion and exclusion criteria, cohort classification, or characterization of patient risks were based on International Classification of Disease, 10th revision (ICD-10) diagnosis and procedure codes and Current Procedural Terminology (CPT) codes.

### Statistical analysis

Patient characteristics and complications were tabulated by catheter cohort within AF type, with means and standard deviations used to summarize continuous variables and counts and percentages to summarize categorical variables.

Logistic regression modeling was employed to determine whether the ablation catheter or type of AF was a significant predictor of the composite procedural complication outcome after adjusting for site procedure volume and any statistically significant differences in patient characteristics. The catheter cohort (ST versus CB2) and AF type (PAF versus PsAF) were included in the model regardless of statistical significance, while stepwise regression was used to select additional covariates with a statistical significance level of 0.05. The patient characteristics considered for inclusion in the model included age, gender, payer type, presence of other arrhythmias (atrial flutter, supraventricular tachycardia, ventricular tachycardia), and comorbidities (cardiomyopathy, chronic kidney disease (CKD), CHF, chronic obstructive pulmonary disease (COPD), diabetes mellitus, history of stroke/TIA, hyperlipidemia, hypertension, obesity, obstructive sleep apnea, vascular disease). Analyses were performed using SAS version 9.4 (SAS Institute, Inc., Cary, NC, USA).

## Results

### Study population

There were 22,013 first AF ablations performed in adult patients within the study period of 2015–2017 that did not also include an exclusionary procedure. Of these ablations, 4620 used an ablation catheter that was identifiable in the data as ST or CB2. The final study population included the subset of 1473 ablations that also recorded the patient’s AF type (1066 in the CB2 cohort and 407 in the ST cohort). The study ablations were contributed by 42 hospitals, with 18.3% performed in an inpatient setting.

The CB2 cohort had a lower percentage of PsAF patients than the ST cohort (38.3% versus 47.2%), so comparisons between cohorts were stratified by AF type (Table 1). The mean age across all patients at the time of their ablation was 63.9 years, which was slightly higher among PsAF patients than PAF patients and similar across catheter cohorts within each AF type. Males comprised 63.1% of the population overall, with more males in the PsAF group than the PAF group (71.2% vs. 57.5%) and in the ST cohort than the CB2 cohort within each AF group (PAF 63.3% vs. 55.6%, PsAF 74.5% vs. 69.6%).

**Table 1:** Patient characteristics at the time of ablation

Characteristic	PAF group			PsAF group		
	CB2 (*N* = 658) *n* (%)	ST (*N* = 215) *n* (%)	*P* value	CB2 (*N* = 408) *n* (%)	ST (*N* = 192) *n* (%)	*P* value
Demographics						
Age, years (mean, SD)	63.2, 10.6	62.7, 12.1	0.5263	65.3, 10.0	64.7, 9.6	0.5100
Male	366 (55.6)	136 (63.3)	0.0494	284 (69.6)	143 (74.5)	0.2192
CHA_2_DS_2_-VASC score			0.1066			0.6021
0	116 (17.6)	47 (21.9)		60 (14.7)	31 (16.1)	
1	164 (24.9)	61 (28.4)		82 (20.1)	52 (27.1)	
2	177 (26.9)	43 (20.0)		107 (26.2)	46 (24.0)	
3	98 (14.9)	28 (13.0)		80 (19.6)	34 (17.7)	
4	53 (8.1)	17 (7.9)		40 (9.8)	16 (8.3)	
5	32 (4.9)	14 (6.5)		22 (5.4)	7 (3.6)	
6	14 (2.1)	3 (1.4)		13 (3.2)	3 (1.6)	
7	3 (0.5)	0 (0.0)		3 (0.7)	2 (1.0)	
8	0 (0.0)	2 (0.9)		1 (0.2)	1 (0.5)	
9	1 (0.2)	0 (0.0)		0 (0.0)	0 (0.0)	
Other types of arrhythmia						
Atrial flutter	188 (28.6)	62 (28.8)	0.9403	111 (27.2)	47 (24.5)	0.4793
Supraventricular tachycardia (SVT)	36 (5.5)	32 (14.9)	< .0001	27 (6.6)	20 (10.4)	0.1062
Ventricular tachycardia	8 (1.2)	5 (2.3)	0.2434	7 (1.7)	3 (1.6)	0.8912
Comorbidities						
Cardiomyopathy	79 (12.0)	20 (9.3)	0.2777	97 (23.8)	39 (20.3)	0.3447
Chronic kidney disease (CKD)	44 (6.7)	20 (9.3)	0.2015	30 (7.4)	13 (6.8)	0.7965
Congestive heart failure (CHF)	121 (18.4)	35 (16.3)	0.4832	141 (34.6)	55 (28.6)	0.1497
Chronic obstructive pulmonary disease (COPD)	105 (16.0)	28 (13.0)	0.2986	76 (18.6)	25 (13.0)	0.0869
Diabetes Mellitus	133 (20.2)	31 (14.4)	0.0590	104 (25.5)	38 (19.8)	0.1255
History of Stroke/TIA	44 (6.7)	18 (8.4)	0.4036	31 (7.6)	14 (7.3)	0.8943
Hyperlipidemia	332 (50.5)	96 (44.7)	0.1394	219 (53.7)	104 (54.2)	0.9105
Hypertension	73 (11.1)	23 (10.7)	0.8718	54 (13.2)	26 (13.5)	0.9180
Obesity	160 (24.3)	43 (20.0)	0.1934	111 (27.2)	36 (18.8)	0.0247
Obstructive sleep apnea	166 (25.2)	45 (20.9)	0.2013	130 (31.9)	52 (27.1)	0.2348
Vascular disease	173 (26.3)	57 (26.5)	0.9493	126 (30.9)	54 (28.1)	0.4918

Comorbidity rates were higher on the whole in the PsAF group when compared with the PAF group, especially cardiomyopathy and CHF rates, which were approximately double in the PsAF group. The most prevalent concurrent arrhythmia was atrial flutter, which did not appear to impact catheter choice, as evidenced by similar rates across cohorts. However, a concurrent diagnosis of supraventricular tachycardia (SVT) did appear to impact catheter choice, as SVT rates were substantially higher in the ST cohort compared with CB2 cohort, both within the PAF and PsAF groups. The most prevalent of all comorbid conditions across the entire population was hyperlipidemia.

### Complications

A total of 97 patients incurred a composite safety event, consisting of any one or more ablation-related complication(s) occurring during the ablation or prior to discharge (Table 2). The unadjusted composite complication rates were similar across catheter cohorts (6.8% CB2, 6.1% ST, *p* = 0.6723). In the PAF group, they were almost identical (6.1% CB2, 6.0% ST, *p* = 0.9862), while they were slightly higher in the CB2 cohort within the PsAF group, but this difference was not statistically significant (7.8% CB2, 6.3% ST, *p* = 0.4858). Patients with complications were more likely to be inpatients than patients without complications (49.5% vs. 16.1%), but the source data does not distinguish between patients that were admitted as inpatients prior to occurrence of a complication versus patients admitted as a consequence of a complication.

**Table 2:** Procedure-related complications occurring prior to ablation discharge

Complication	PAF group			PsAF group		
	CB2 *N* = 658 *n* (%)	ST *N* = 215 *n* (%)	*P* value	CB2 *N* = 408 *n* (%)	ST *N* = 192 *n* (%)	*P* value
Composite complication outcome	40 (6.1)	13 (6.0)	0.9862	32 (7.8)	12 (6.3)	0.4850
Individual complications						
Tamponade/pericardial events	12 (1.8)	4 (1.9)	0.9722	9 (2.2)	2 (1.0)	0.3214
Pericardial drainage procedure	4 (0.6)	0 (0.0)	0.2519	2 (0.5)	1 (0.5)	0.9604
Respiratory complications	15 (2.3)	0 (0.0)	0.0255	7 (1.7)	4 (2.1)	0.7542
Stroke or other cerebral/pre-cerebral occlusion/stenosis	10 (1.5)	3 (1.4)	0.8960	6 (1.5)	3 (1.6)	0.9311
Vascular access events	5 (0.8)	5 (2.3)	0.0611	5 (1.2)	3 (1.6)	0.7371
Hemorrhage/blood transfusion	8 (1.2)	2 (0.9)	0.7326	3 (0.7)	2 (1.0)	0.7002
Phrenic nerve complication	2 (0.3)	0 (0.0)	0.4183	2 (0.5)	0 (0.0)	0.3312
Myocardial infarction	1 (0.2)	1 (0.5)	0.4044	1 (0.2)	0 (0.0)	0.4924
Pulmonary embolism	1 (0.2)	0 (0.0)	0.5674	0 (0.0)	1 (0.5)	0.1446

**Fig. 1:**
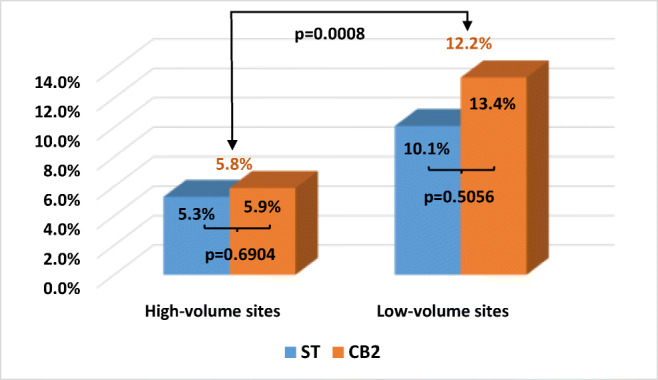
Acute complications across high- and low-volume centers. A total of 5.8% of patients ablated at high-volume sites had an acute procedure-related complication versus 12.2% of patients ablated at low-volume sites (*p* = 0.0008). Complication rates did not vary significantly by catheter cohort within the high-volume sites (5.9% CB2 vs. 5.3% ST, *p* = 0.6904) or within the low-volume sites (13.4% CB2 vs. 10.1% ST, *p* = 0.5056)

The number of ablations performed by a site had an impact on complication rates, with lower rates in sites that performed more ablations. After testing cutoffs of 10, 20, 30, and 40 procedures for statistical power, a high-volume site was defined as one with 20 or more ablations that met all inclusion and exclusion criteria for the study. Using this definition, 5.8% of patients ablated at high-volume sites had an acute procedure-related complication versus 12.2% of patients ablated at low-volume sites (*p* = 0.0008) (Fig. 1). Complication rates did not vary significantly by catheter cohort within the high-volume sites (5.9% CB2 vs. 5.3% ST, *p* = 0.6904) or within the low-volume sites (13.4% CB2 vs. 10.1% ST, *p* = 0.5056).

After adjusting for site procedure volume and significant patient characteristics with logistic regression, neither the ablation catheter nor AF type had a significant impact on the occurrence of an acute procedure-related complication (Table 3). CKD and increasing age were the patient characteristics most strongly associated with an event, followed by COPD, CHF, obesity, and ventricular tachycardia, respectively.

**Table 3:** Logistic regression model of composite acute ablation-related complication

Patient characteristic	Odds ratio [95% confidence interval]	*P* value
Primary Predictors of Interest		
Type of catheter ablation: ST versus CB2	0.86 [0.53, 1.41]	0.5544
Type of AF: PsAF versus PAF	1.08 [0.70, 1.67]	0.7376
Additional Predictors with Significance at 0.05 Level		
Chronic kidney disease (CKD)	2.81 [1.59, 4.94]	0.0003
Age, per year of increase	1.04 [1.01, 1.06]	0.0019
Ablation at site with ≥ 20 ablations in study	0.45 [0.26, 0.75]	0.0024
Chronic obstructive pulmonary disease (COPD)	1.83 [1.12, 2.98]	0.0153
Congestive heart failure (CHF)	1.70 [1.07, 2.70]	0.0260
Obesity	1.66 [1.03, 2.66]	0.0370
Ventricular tachycardia	2.99 [0.99, 9.08]	0.0529

## Discussion

Complications following catheter ablation for AF adversely impact patient health, increase hospital costs, and negatively influence patient satisfaction and quality of life. As a result, it is important to understand the impact, if any, that the latest technologies and ablation methodologies will have on the incidence of complications. It is also important to understand which patients will be at the greatest risk of complication. In the current study, patients undergoing AF ablation with two of the latest catheter technologies had similarly low rates of acute ablation-related complications (ST 6.1%, CB2: 6.8%, *p* = 0.6723). After adjusting for site volume and patient characteristics in a logistic regression model, neither the catheter nor the type of AF was statistically significantly associated with the occurrence of a complication. The most predictive patient characteristics, in order of association with complications, were CKD, age, COPD, CHF, obesity, and ventricular tachycardia. Sites that performed a higher volume of ablations also had significantly lower complication rates than sites with lower volumes.

In the nationally representative National Inpatient Sample (NIS) database, comprising 93,801 AF procedures performed between 2000 and 2010, the incidence of AF ablation-related complications was 6.5% [11]. The current study found a similar incidence of complications in both the ST and CB2 catheter cohorts, with 6.1% and 6.8% respectively. The most common complications in the NIS database study were combined cardiac events (2.5%), followed by vascular events (1.5%), respiratory events (1.3%), and neurologic events (1.0%), which is roughly similar to the event categories and rates reported in the current study. It should be noted that the NIS study included a 0.46% death rate for patients ablated in an inpatient setting. While our data did not allow identification of in-hospital death due to patient de-identification algorithms used by the data vendor, we would expect deaths to have a much smaller impact on our rates, due to the fact that only 18.3% of our ablations were inpatient procedures and also due to the later timeframe of our study and the improvement in the safety of AF ablation in recent years. Complications in the NIS study were associated with physicians who performed < 25 ablations annually and hospital volumes of < 40 procedures annually, which parallels our finding that a site procedure volume of at least 20 ablations meeting our inclusion criteria was associated with a significant reduction in complication rates.

Another study used the Premier Healthcare Database, a US hospital database similar to the one used in the current study, to compare 1261 RF ablations with 1276 CB2 ablations [12]. Though focused on cost, the study also reported similar complication rates in RF and CB2 cohorts after adjustment for significant patient and hospital characteristics (*p* values, 0.4888 and 0.5072 for ablations performed in the inpatient and outpatient settings). The complication endpoint in this study was constructed very similarly to the one used in the current study. However, the prior study included ablations performed in 2013–2014; thus, the RF cohort would have been comprised almost exclusively of non-CF catheters due to the 2014 FDA approval of ST.

Procedure-related complications for ST and CB2 ablations have been reported in several prospective interventional studies. The first to specifically compare these particular catheters is currently underway and recently reported on the initial results, including periprocedural complication rates [13, 14]. As in the current study, the ST cohort had a non-significantly lower rate of events than the two CB2 cohorts (2.6% vs. 5.2% and 6.0%, *p* = 0.24). In addition, the prospective multicenter SMART-AF trial enrolled 161 PAF patients in a single ST ablation cohort, reporting 2.5% tamponade, 1.9% pericarditis, and 2.5% vascular complication rates within 7 days of the procedure [15]. There were no cases of atrioesophageal fistula, PV stenosis, thromboembolism, cerebrovascular accident, MI, stroke, or death during the 12 months following the procedure. Among 762 patients randomized to RF or cryoballoon ablation in the FIRE and ICE study, the primary composite safety endpoint was not significantly different through 12 months (HR, 0.78, 95% CI, (0.52, 1.18)) [16]. However, this trial had an imbalance of 75.6% CB2 catheters (vs. first generation CB) compared to only 24.7% CF catheters (vs. non-CF RF). Groin complications (1.0% CB, 2.1% RF) and phrenic nerve injury (1.3% in CB only) were the most common complications in this study, with no atrioesophageal fistula, pulmonary vein stenosis, or procedure-related death.

Additional non-randomized clinical studies, primarily utilizing smaller samples of consecutive ablations performed at a single site, have also compared ST vs. CB2 [6–8, 17–19]. None of these studies found a statistically significant difference in complication rates between catheter cohorts, though none were powered to do so. Several studies did report that the profiles of events differed in some respect. In particular, three studies reported that phrenic nerve palsy was exclusively seen in the CB2 arms [6, 17, 19], while two reported that tamponade was only seen in the ST arms [6, 19]. Another three of the studies reported less fluoroscopy exposure in the ST arms [7, 8, 18], which is a safety consideration that could not be studied in the current hospital database analysis due to the lack of procedural detail reported in administrative databases.

While complication rates are commonly reported in studies of AF ablation, the patient characteristics associated with risk of experiencing an ablation-related complication are less studied. The current study was comprised of 1473 ablations, 97 of which resulted in complications. This sample size was sufficient to identify 6 baseline patient characteristics that were significantly associated with complications at a level of approximately 0.05 or below. Despite some differences in the specific characteristics that were measured across the two studies, both the current study and a 2016 study by Padala et al. identified CHF, CKD/renal disease, COPD, and age as important risk factors for acute ablation-related complications [20]. It is well-known that CHF and age are among the greatest risk factors for stroke in an AF population, as commonly measured via the CHA_2_DS_2_-VASc risk score, [21] and similarly that age and CKD increase bleeding risk, as per the HAS-BLED risk score [22, 23]. Presumably, COPD would increase the risk of respiratory complications.

### Limitations

The primary limitations of this study are the retrospective design and the use of administrative hospital data, which does not provide the level of clinical detail afforded by prospectively designed clinical studies. Patients could not be followed beyond their ablation discharge, thus limiting the capture of late complications or clinical effectiveness. In addition, the use of administrative hospital data did not allow inclusion of procedural details such as fluoroscopy usage, complete pre-ablation medical and medication histories, death, or clinical details such as the severity of comorbidities. These unknowns could potentially confound or bias the results. However, the baseline characteristics that were captured at the time of the procedure and are known to be risk factors in an AF population were similar across catheter cohorts. Another unmeasured patient characteristic that could impact safety outcomes is oral anticoagulation (OAC) strategy. However, if OAC is used per the most recent guidelines, it would not be expected to differ across cohorts. Moreover, our comparison only requires that the overall population ablated with ST is not systemically anticoagulated differently than the overall population ablated with cryoablation, which we believe is a very reasonable assumption.

Another limitation of any retrospective study design is that the sample size is dictated by the data that is available from the source that meets all inclusion criteria. Thus, the sample size is not chosen prospectively to correspond to the statistical power required for testing of hypotheses. Without statistically powered hypotheses, a lack of statistical significance (i.e., high *p* values) does not imply a lack of clinical significance. Therefore, any observed differences in effect sizes that are not statistically significant but appear to be of clinically significant magnitude will require further studies to confirm.

Text mining algorithms used to define the catheter cohorts were limited to hospitals that recorded a sufficient description of the ablation catheter, resulting in exclusion of ablations that may have otherwise met the inclusion criteria. In particular, in many cases, it was impossible to distinguish among manufacturers or generations of RF catheters, likely resulting in a more complete capture of the CB2 cohort versus an underrepresentation of the ST cohort. Also, the ST cohort included two generations of contact force catheter because the data available at the start of the study did not yet include sufficient quantity of ablations using the newer porous cooling tip design. Thus, any safety benefits attributable to the new cooling tip design could not be studied.

Despite the limitations of the retrospective study design and the inherent shortcomings of administrative data, the current study has several unique strengths. First and foremost, the large and nationally representative population enabled us to present a highly generalizable picture of current clinical practice in the USA. Unlike most clinical trials or single-site studies of consecutive patients, this data reflects a combination of high-volume and low-volume sites and thus is more reflective of the full spectrum of AF ablation treatment in the USA. The importance of this site variation is reflected in the finding that sites performing low volumes of ablations had complication rates more than double the rates seen in sites performing higher volumes. In addition to the benefits of a diverse population, the large sample size allowed for stratification of complications by both catheter and AF type and also for identification of patient characteristics that are associated with a higher risk of experiencing an acute ablation-related complication.

## Conclusions

This large real-world study found a slightly lower but not statistically different rate of complications in AF ablations performed with ST compared with CB2. Site volume was an important predictor of complication rates, as was patient age and several baseline comorbid conditions. AF type was not a significant predictor after adjusting for site volume and patient characteristics. This evidence fills a gap in the understanding of ablation-related complication rates with the latest ablation technologies as well as a comparison between the latest generations of RF and cryoballoon catheter technologies for AF ablation in a large real-world US population.
